# A Coupled Finite Element and Crystal Plasticity Study of Friction Effect on Texture Evolution in Uniaxial Compression of NiTi Shape Memory Alloy

**DOI:** 10.3390/ma11112162

**Published:** 2018-11-01

**Authors:** Li Hu, Shuyong Jiang, Tao Zhou, Qiang Chen

**Affiliations:** 1 College of Material Science and Engineering, Chongqing University of Technology, Chongqing 400054, China; huli@cqut.edu.cn (L.H.); zt19811118@cqut.edu.cn (T.Z.); 2 College of Mechanical and Electrical Engineering, Harbin Engineering University, Harbin 150001, China; 3 Southwest Technology and Engineering Research Institute, Chongqing 400039, China; 2009chengqiang@163.com; 4 Precision Forming Integrated Manufacturing Technology of Collaborative Innovation Center, Chongqing 400039, China

**Keywords:** NiTi shape memory alloy, texture evolution, deformation mechanism, finite element method, crystal plasticity

## Abstract

A coupled macro-meso-scale numerical simulation is applied to investigate the friction effect on texture evolution during uniaxial compression of NiTi shape memory alloy at 400 °C. In this approach, macroscale finite element simulations in consideration of various friction coefficient are conducted and then the corresponding velocity gradients in various regions are extracted mainly based on the delivery deformation gradient in the user-defined material subroutine (UMAT) in ABAQUS code. These velocity gradients are regarded as the deformation conditions applied in the mesoscale VPSC model. Simulation results in terms of macroscale finite element modeling demonstrate that only within the region of minimum deformation zone which is close to the die, friction effect has a nonnegligible influence on the velocity gradient. Simulation results with respect to the mesoscale VPSC modeling show that the affine and Neff = 10 linearization schemes provide the best predictions for NiTi shape memory alloy with cubic structure. Furthermore, the friction effect does have an influence on the evolution of slip mode activities in various deformation zones and therefore results in the inhomogeneous texture evolution within the deformed sample during uniaxial compression.

## 1. Introduction

The frictional behavior between a workpiece and forming tool is unavoidable during the real-world plastic deformation conditions, as Tabor in 1951 had proposed that there still exists a measurable coefficient of friction ranging from 0.1 to 0.15 between highly polished metallic surfaces and diamond [1]. In addition, the frictional behavior during plastic deformation has a significant influence on the dimensional accuracy and the emergence of defects of the finally manufactured products. More importantly, the frictional behavior during the bulk-metal forming process plays an important role in the evolution of crystallographic texture within the manufactured products, which in turn can modify the corresponding physical and mechanical properties [2]. 

Numerous experimental methods and simulation tools have been proposed to determine such friction effect on the texture evolution of metallic materials during various plastic deformation process, including nano-indentation [3], cold-rolling [4], plane strain compression [5], equal channel angular pressing [2,6] and so on. It is worth noting that texture predictions conducted in the above investigations use one of the following three numerical models, including the crystal plasticity finite element (CPFE) model, the visco-plastic self-consistent (VPSC) model and the Taylor model. Although the aforementioned studies have confirmed that the existence of friction coefficient contributes to the spatial variation in texture during plastic deformation, a thorough analysis with respect to the deformation mechanisms underneath the texture evolution in consideration of the friction effect is still unavailable to our present knowledge. 

To date, there still exists certain restrictions in terms of revealing the plastic deformation mechanisms of metallic materials under simple deformation conditions, including uniaxial compression/tension, simple shear, and plane strain compression, via experimental measurements [7]. Therefore, numerical methods, particularly the crystal plasticity-based models including VPSC model and CPFE model, become perfect candidates for clarifying the plastic deformation mechanisms and the corresponding texture evolution during plastic deformation. It is generally accepted that the friction effect during plastic deformation contributes to complicating the distribution of deformation within metallic materials [8,9]. Hence, the keys to successfully apply the crystal plasticity-based models lie in the accurate extraction of the realistic deformation history in consideration of the friction effect. As for the CPFE model, Hu et al. [10] have proposed a method based on the sub-model technique of the finite element code ABAQUS to extract the deformation history in the region of interest. Although this method is effective, it is not quite straightforward and relatively complicated during application. With respect to the VPSC model, it is widely used in association with simple monotonic loading conditions including uniaxial compression/tension [11,12]. To analyze texture evolution during non-monotonic deformation resulting from the friction effect, a corresponding deformation history should be obtained and used as input during numerical simulation. Li et al. [2] have successfully presented a framework for obtaining local deformation history of a deformation process using ABAQUS code in a format readable by VPSC model. This method about extracting deformation history endows VPSC model with the capability in dealing with the complicated deformation history derived from the friction effect. To our knowledge, there is no literature which clearly sheds a light on the deformation mechanisms superimposed by the friction effect.

In the present study, texture evolution of NiTi shape memory alloy undergoing uniaxial compression at 400 °C is investigated in consideration of the friction effect via VPSC model. Firstly, the needed material parameters for NiTi shape memory alloy are calibrated by means of the particle swarm optimization (PSO) algorithm [13]. Subsequently, the macroscale finite element simulations using ABAQUS code are conducted with various coefficient of friction and the corresponding deformation history is then extracted as input for VPSC model. It is noted that in the present study the first VPSC modeling study is implemented by using the complicated deformation conditions resulting from the friction effect during uniaxial compression of NiTi shape memory alloy at 400 °C.

## 2. Simulation Procedures 

### 2.1. VPSC Modeling

In the present study, texture evolution during uniaxial compression of NiTi shape memory alloy at 400 °C is simulated via VPSC model, which is developed by Lebensohn et al. [14]. VPSC stands for the visco-plastic self-consistent and refers to the particular mechanical regime highlighted by VP and to the approach termed as SC. In this model, plastic deformation of polycrystalline aggregate as well as the global mechanical response can be simulated in consideration of the full anisotropy of properties and responses of the constituting individual grains. To achieve the aforementioned targets, each grain is treated as an ellipsoidal visco plastic inclusion associated with a volume fraction. Furthermore, each grain is assumed to be embedded in and interact with a homogeneous effective medium (HEM), which represents the ‘average’ environment seen by each grain. As for NiTi shape memory alloy adopted in the present study, plastic deformation at 400 °C is only based on dislocation slip without the assistance of deformation twinning and phase transformation [13,15]. Hence, plastic deformation is accommodated by the {110} <100> slip mode, {010} <100> slip mode and {110} <111> slip mode [16,17]. 

For the slip-based deformation, the shear rate ${\dot{\gamma }}^{\alpha }$ is associated with the resolved shear stress $\tau^{\alpha }$ and the critical resolved shear stress (CRSS) $\tau_{c}^{\alpha }$ in each slip system α.

(1) $${\dot{\gamma }}^{\alpha }={\dot{\gamma }}_{0}{\left|\frac{\tau^{\alpha }}{\tau_{c}^{\alpha }}\right|}^{\frac{1}{m}}\mathrm{sgn}(\tau^{\alpha }),$$

where ${\dot{\gamma }}_{0}$ stands for the reference shear strain rate and its value is determined to be 0.001 s^−1^ in consideration of experimental loading rate. The parameter m qualifies the strain rate sensitivity of material and its value is chosen to be 20 indicating a certain rate sensitivity of NiTi shape memory alloy [18]. sgn(.) returns an integer indicating the symbol of an argument (.).

The CRSS in each slip system α evolves by the following extended Voce law.

(2) $$\tau_{c}^{\alpha }=\tau_{0}^{\alpha }+(\tau_{1}^{\alpha }+\theta_{1}^{\alpha }\Gamma )(1-\exp(-\frac{\theta_{0}^{\alpha }}{\tau_{1}^{\alpha }}\Gamma )),$$

where Γ stands for the total accumulated shear strain $\Gamma =\sum_{s}\Delta \gamma^{s}$ on all activated slip systems. $\tau_{0}^{\alpha }$, $\theta_{0}^{\alpha }$, $\tau_{0}^{\alpha }+\tau_{1}^{\alpha }$ and $\theta_{1}^{\alpha }$ are the initial CRSS, the initial hardening rate, the back-extrapolated CRSS and the asymptotic hardening rate, respectively. In the present study, all the self and latent hardening parameter are set to be 1 as only dislocation slip is evolved in the plastic deformation. 

### 2.2. Identification of Material Parameters

**Figure 1:**
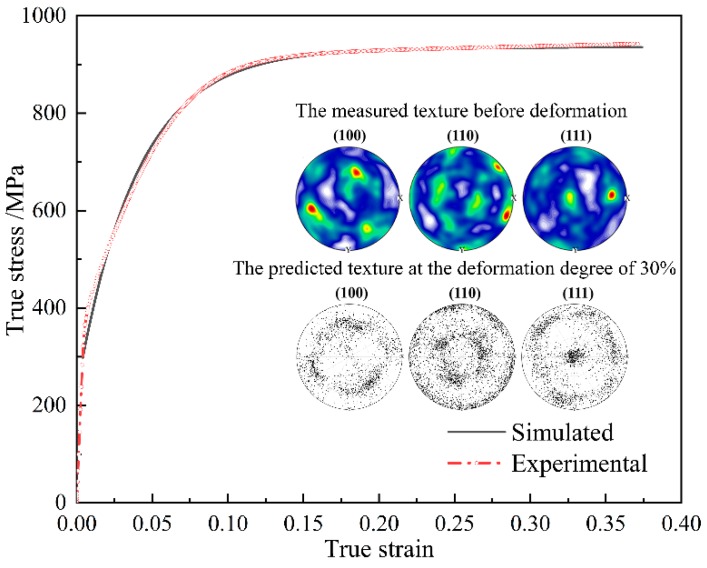
The simulated stress-strain curve and texture at the deformation degree of 30% based on the 1000 discrete orientations and the corresponding experimental results.

To simulate uniaxial compression along the normal direction with VPSC model, the component of velocity gradient $L_{\mathrm{zz}}$ is imposed and it is equivalent to the strain rate along the loading direction. Moreover, the components of stress tensor $\sigma_{\mathrm{xx}}$ and $\sigma_{\mathrm{yy}}$ are set to be zero to allow for ovalization of the deformed sample. In fact, the uniaxial compression of NiTi shape memory alloy at 400 °C is conducted at the constant strain rate of 0.001 s^–1^. It is important to note that a heating device is applied to heat both the specimen and dies to 400 °C before loading. In other words, NiTi shape memory alloy specimen is always to be subjected to compression deformation at a constant temperature of 400 °C. This operation is to confirm the isothermal condition during uniaxial compression. Then the measured stress-strain curve shall not only be used to fit the material parameters in the VPSC model at the mesoscale but also used to obtain the elastic and plastic data which are needed during the finite element modeling at the macroscale. In addition, the applied grain orientations in the VPSC model are derived from the measured texture of the adopted NiTi shape memory alloy using the electron back-scattered diffraction (EBSD) equipment, as shown in Figure 1. The method about extracting 1000 discrete orientations is based on discretizing the orientation distribution function (ODF) via the toolbox MTEX in MATLAB code. The previous work conducted by Hu et al. has confirmed the validity of this procedure [7]. The detailed contents about the sample preparation and the observation equipment applied in the mechanical experiment and EBSD experiment can be specified in [7,13], and therefore they shall be declared here. 

Chapius et al. [11] have confirmed that the adopted material parameters in the VPSC model have a significant influence on the plastic deformation of metallic material as well as the corresponding global mechanical response. Unlike the frequently used “trial-error” method which optimizes the numerical results by virtue of the optical observation on the difference between the numerical stress-strain curve and the experimental one, in the present study the calibration of material parameters is realized via a PSO algorithm with high efficiency and accuracy [13]. In PSO algorithm, the member of all population keeps constant during the entire process of optimization and their interactions iteratively contribute to the improvement of the quality about problem solutions by virtue of tracking the personal best (pBest) and global best (gBest) in each iteration. A more detailed description of PSO algorithm can be found in [13,19]. Furthermore, it is worth noting that there are various candidate linearization schemes in the VPSC model, which obviously influence the plastic deformation and texture evolution of metallic material. The previous researches have confirmed that the affine scheme gives the best accordance between simulations and experiments for face centered cubic (FCC) material [20] and hexagonal close packed (HCP) material [21]. As for the adopted NiTi shape memory alloy with a cubic structure at 400 °C, firstly the affine scheme is assumed to the optimized one employed in the present study, and then an assessment of the predictive capabilities of various linearization schemes is conducted. 

Based on the aforementioned PSO algorithm, the optimized material parameters are obtained and shown in Table 1. Then the uniaxial compression of NiTi shape memory alloy at the deformation of 30% is simulated and the corresponding mechanical response and texture evolution are shown in Figure 1. It can be seen from Figure 1 that the experimental stress-strain curve is in good accordance with the predicted one. Moreover, the simulated pole figures show an obvious <111> fiber texture which is very akin to the reported texture measured by EBSD experiment and texture predicted by virtue of CPFE simulation [13]. These observations confirm the validity of the adopted material parameters used in the VPSC model.

**Figure 2:**
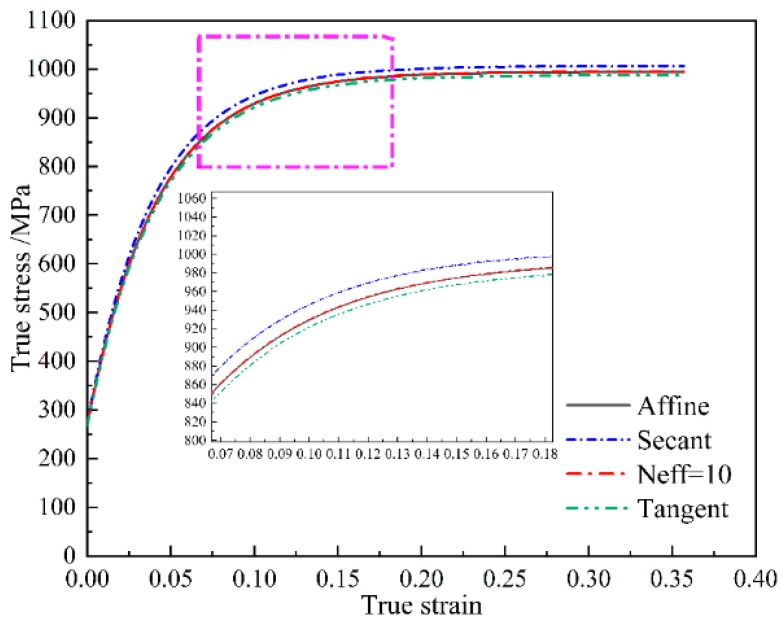
The simulated stress-strain curves in the case of various linearization schemes in the VPSC model during uniaxial compression.

**Figure 3:**
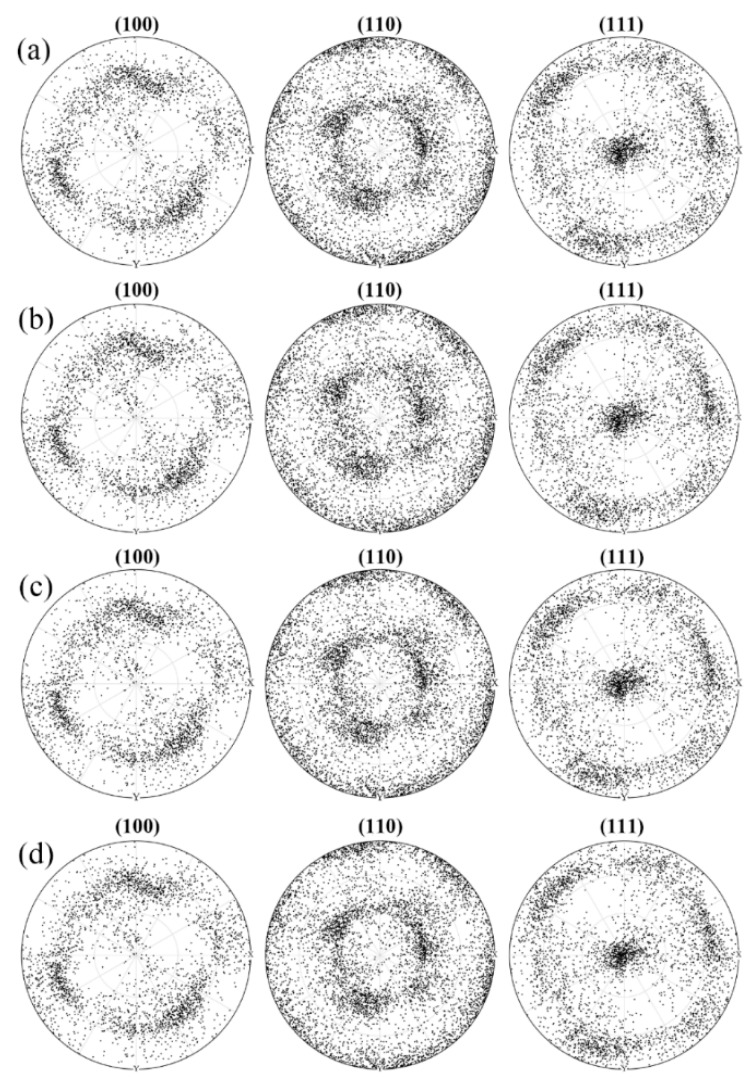
The simulated pole figures at the deformation degree of 30% in the case of various linearization schemes in the VPSC model: (**a**) Affine scheme; (**b**) Secant scheme; (**c**) Neff = 10 scheme; (**d**) Tangent scheme.

**Figure 4:**
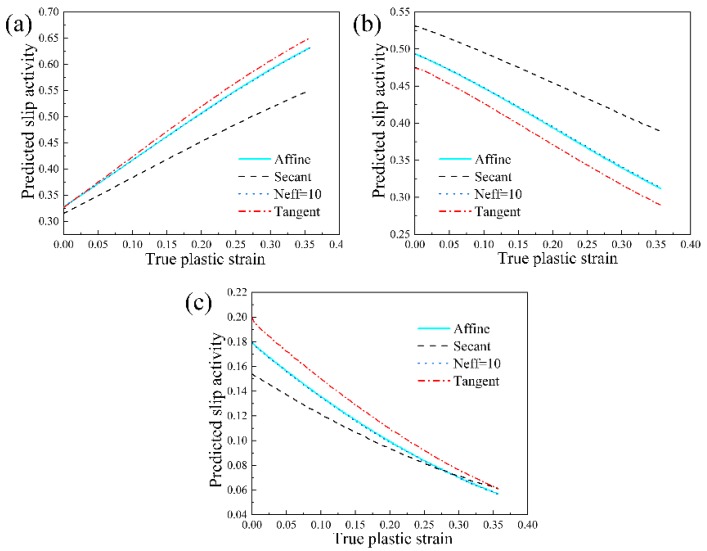
The simulated slip mode activities at the deformation degree of 30% in the case of various linearization schemes in the VPSC model: (**a**) {110} <100> slip mode; (**b**) {110} <111> slip mode; (**c**) {010} <100> slip mode.

Based on the identified material parameters as shown in Table 1, a further validation is given in the following with respect to the predictive capabilities of various linearization schemes. Figure 2 shows the simulated stress-strain curves in the case of various linearization schemes under uniaxial compression. All linearization schemes produce a reasonable mechanical response. However, a noticeable difference in the predicted stress-strain curves is that the affine scheme and Neff = 10 scheme generate an in-between mechanical response, as compared to the Secant scheme and Tangent scheme. Figure 3 shows the simulated texture at the deformation degree of 30%. All the linearization schemes in the VPSC model can capture the main feature in the measured deformed texture with a <111> fiber texture component [13]. This observation indicates that the texture prediction is independent to the applied linearization scheme for the adopted NiTi shape memory alloy. In addition, the predicted slip mode activities with respect to {110} <100> slip mode, {110} <111> slip mode and {010} <100> slip mode in the case of various linearization schemes are given in Figure 4. All linearization schemes produce a similar evolution law in terms of various slip modes that the activity with respect to {110} <100> slip mode possesses a continuous increasing character during plastic deformation, while the activities in terms of {110} <111> slip mode and {010} <100> slip mode tend to decrease with the increasing plastic deformation. It is interesting to find that the affine scheme and Neff = 10 scheme also give a medial simulation result for the predicted slip mode activities. Consequently, based on the aforementioned assessments from different perspectives including mechanical response, texture evolution and slip mode activity, the affine scheme and Neff = 10 scheme give the best predictions, demonstrating the validity of employed affine scheme in the present study for NiTi shape memory alloy with cubic structure. 

### 2.3. Finite Element Modeling

Although these simple deformation conditions can be easily conducted in the VPSC simulations in terms of the velocity or displacement gradients, including uniaxial compression/tension, plane strain compression and simple shear, the deformation history in consideration of the friction effect is rather complicated and would be implemented with difficulty in the simulations using VPSC model. Hence, finite element modeling counting on the friction effect during plastic deformation is needed to extract the deformation history in some selected elements. In the present study, the method proposed by Li et al. [2] and Latypov et al. [22] is applied to calculate the corresponding deformation history in various regions of a deformed sample during uniaxial compression. 

In continuum mechanics, the velocity gradient tensor $L_{t}$ at the beginning of a time increment step can be calculated from the total deformation gradient $F_{t}$ as follows:

(3) $$L_{t}={\dot{F}}_{t}F_{t}{}^{-1}$$

By using a Taylor expansion, the total deformation gradient $F_{T}$ at the end of a time increment step is calculated by the following equation.

(4) $$F_{T}\approx (I+\Delta tL_{T})F_{t}$$

where Δt refers to a time increment Δt=T−t. I stands for the second order identity tensor. By reforming Equation (4), $L_{T}$ could be calculated by

(5) $$L_{T}=\frac{F_{T}F_{t}{}^{-1}-I}{\Delta t}$$

The aforementioned equations in terms of calculating the velocity gradient can be readily implemented in the user-defined material subroutine (UMAT) in ABAQUS code, in which ABAQUS code passes Δt, $F_{t}$ and $F_{T}$ at the beginning of each time increment step [23]. 

**Figure 5:**
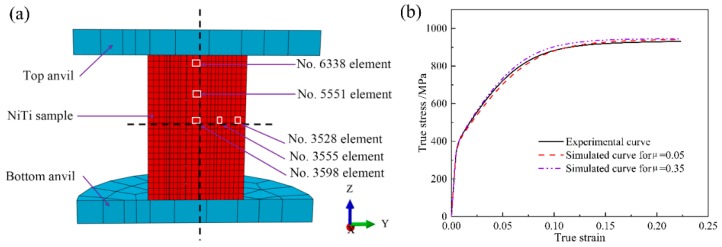
(**a**) Schematic diagram of the finite element modeling of uniaxial compression of NiTi shape memory alloy via ABAQUS code. (No. 6338 element is in the minimum deformation zone. No. 5551 element is located between the minimum deformation zone and the principal deformation zone. No. 3598 element is in the principal deformation zone. No. 3555 element is located between the principal deformation zone and the intermediate deformation zone. No. 3528 element is in the intermediate deformation zone.); (**b**) True stress-strain curves with respect to the experimental measurement and finite element modeling in the case of friction coefficients of 0.05 and 0.35 at the deformation degree of 20%.

As for finite element modeling of uniaxial compression at a constant strain rate of 0.001 s^−1^ in consideration of friction effect, as shown in Figure 5a. Finite sliding with tangential behavior is defined for the surface-to-surface contact between the dies and sample with friction coefficient of 0.05 and 0.35. In addition, all dies and sample are meshed with the eight-node brick elements with reduced integration (C3D8R). Figure 5b shows the comparison between the experimentally measured true stress-strain curve and the predicted ones at the deformation degree of 20% via finite element modeling at the macroscale. It can be seen from Figure 5b that there only exists a minor difference between the experimental result and the simulates ones, which demonstrates that the friction has a very small influence on the global mechanical response during uniaxial compression. This observation confirms that finite element modeling at the macroscale is a valid method. The convergence analysis with respect to the mesh density can be found in Appendix A. It confirms the validity of meshing NiTi shape memory alloy specimen with 6696 C3D8R elements in the present study. It is generally accepted that there are three different deformation zones in the deformed sample during uniaxial compression, namely the minimum deformation zone at the top and bottom part of the deformed sample close to the dies, the principal deformation zone in the central of the deformed sample and the intermediate deformation zone in the lateral part of the deformed sample. The generation of these three deformation zones is derived from the friction effect. Hence, after finite element simulation, deformation history in various elements as shown in Figure 5 is extracted to investigate the friction effect on the evolution of texture during uniaxial compression.

## 3. Results and Discussion

### 3.1. Texture Evolution in Minimum Deformation Zone and Principal Deformation Zone

**Figure 6:**
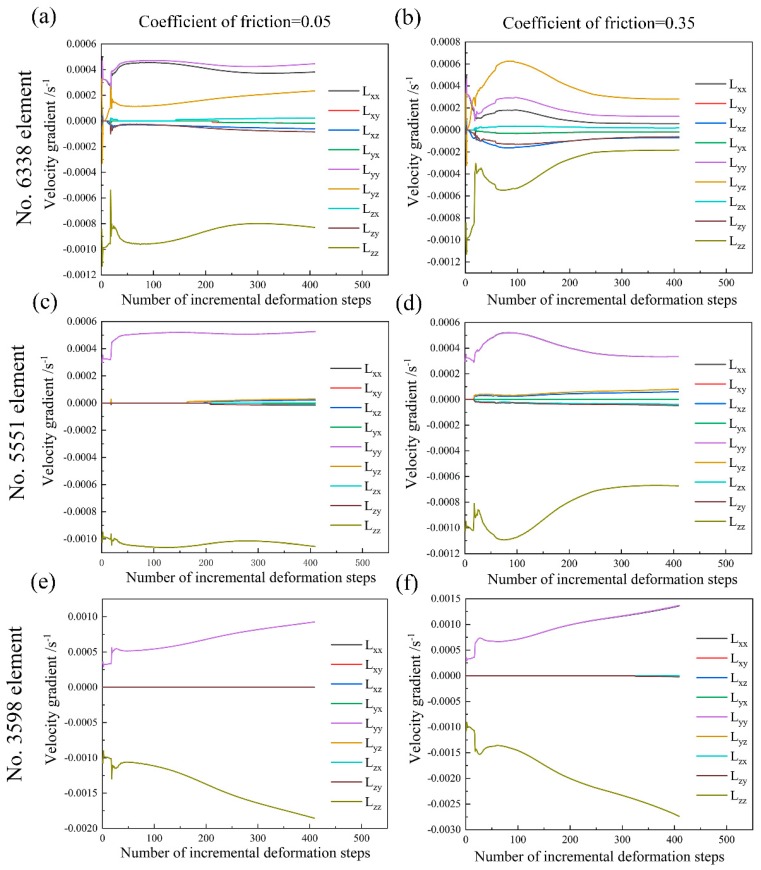
Velocity gradient in No. 6338 element, No. 5551 element and No. 3598 element during uniaxial compression at the deformation degree of 20% with: (**a**,**c**,**e**) Friction coefficient of 0.05; (**b**,**d**,**f**) Friction coefficient of 0.35.

**Figure 7:**
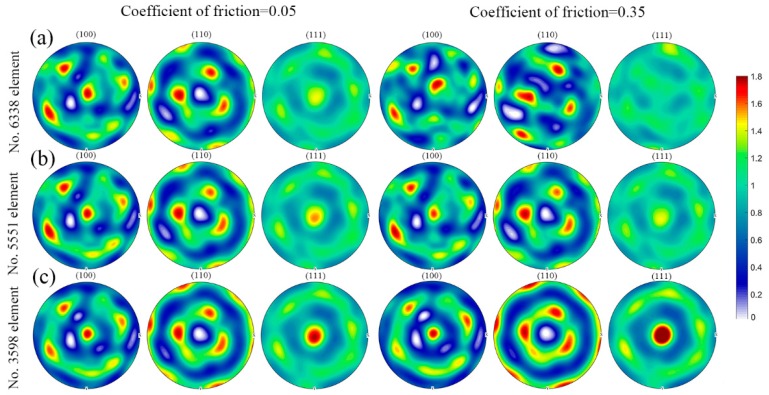
Predicted pole figures with respect to various elements in the case of friction coefficient of 0.05 and 0.35 during uniaxial compression at the deformation degree of 20%: (**a**) No. 6338 element; (**b**) No. 5551 element; (**c**) No. 3598 element. The corresponding color code represents the pole intensity (multiples of random distribution).

Figure 6 shows the deformation history in various elements during uniaxial compression at the deformation degree of 20% in the case of friction coefficient of 0.05 and 0.35. These selected elements, namely No. 6338 element, No. 5551 element and No. 3598 element, refer to various regions within the deformed sample ranging from the minimum deformation zone to the principal deformation zone. The velocity gradient extracted from the element locating in the minimum deformation zone (No. 6338 element) is more sensitive to the friction coefficient as compared to the element located in between the minimum deformation zone and the principal deformation zone (No. 5551 element) and the element locating in the principal deformation zone (No. 3598 element). In addition, the velocity gradient with respect to No. 3598 element shows a minor difference in the case of friction coefficient of 0.05 and 0.35. This observation verifies that the friction coefficient does have an obvious influence on the plastic deformation in the minimum deformation zone close to the dies and its effect rapidly decreases along with the increasing distance to the surface of deformed sample close to the dies. Using these extracted velocity gradients in Figure 6 as the input of deformation history in the VPSC model, texture evolution in these various regions is predicted, as shown in Figure 7 with the (100), (110) and (111) pole figures. It is obvious that except for the pole figures with respect to No. 6338 element with friction coefficient of 0.35, all the simulated textures are mainly composed of <111> fiber texture components during uniaxial compression. This observation is akin to the measured texture evolution during uniaxial compression of NiTi shape memory alloy reported in [7]. Moreover, the intensity of <111> fiber texture component gradually increases from the minimum deformation zone to the principal deformation zone. This phenomenon is associated with various deformation history affected by the friction coefficient, as shown in Figure 6. 

### 3.2. Texture Evolution in Principal Deformation Zone and Intermediate Deformation Zone

**Figure 8:**
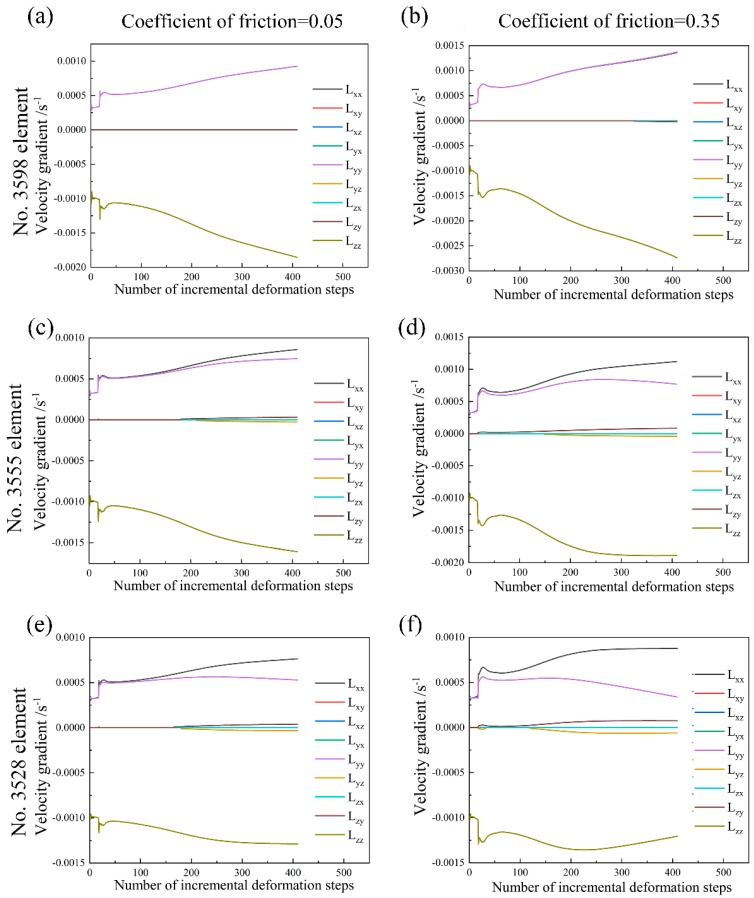
Velocity gradient in No. 3598 element, No. 3555 element and No. 3528 element during uniaxial compression at the deformation degree of 20% with: (**a**,**c**,**e**) Friction coefficient of 0.05; (**b**,**d**,**f**) Friction coefficient of 0.35.

**Figure 9:**
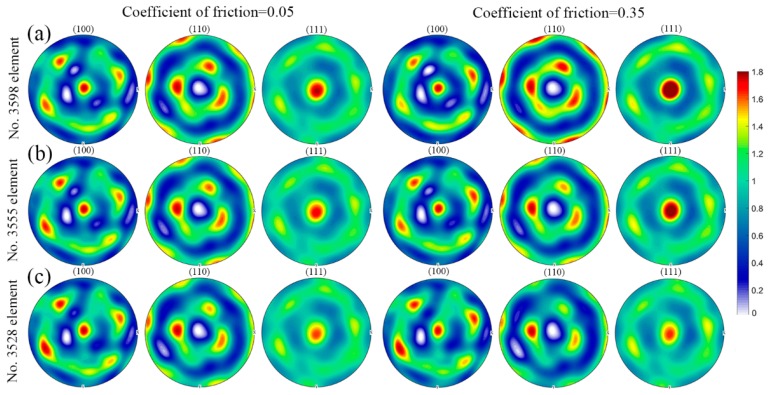
Predicted pole figures with respect to various elements in the case of friction coefficient of 0.05 and 0.35 during uniaxial compression at the deformation degree of 20%: (**a**) No. 3598 element; (**b**) No. 3555 element; (**c**) No. 3528 element. The corresponding color code represents the pole intensity (multiples of random distribution).

Figure 8 shows the deformation history in the element within the principal deformation zone (No. 3598 element), the element located in between the principal deformation zone and the intermediate deformation zone (No. 3555 element) and the element within the intermediate deformation zone (No. 3528 element) during uniaxial compression at the deformation degree of 20% in the case of friction coefficient of 0.05 and 0.35. The corresponding texture evolution in these elements is shown in Figure 9. It can be seen from Figure 8 that the friction effect shows relatively bigger influence on the components of velocity gradient, namely $L_{\mathrm{xx}}$ and $L_{\mathrm{yy}}$ as compared to other components. However, the total change of velocity gradient is rather small in these three selected elements. Consequently, the friction effect on the texture evolution is also small, as shown in Figure 9. In addition, the intensity of <111> fiber texture component gradually decreases from the principal deformation zone to the intermediate deformation zone.

### 3.3. Deformation Mechanisms under Uniaxial Compression

**Figure 10:**
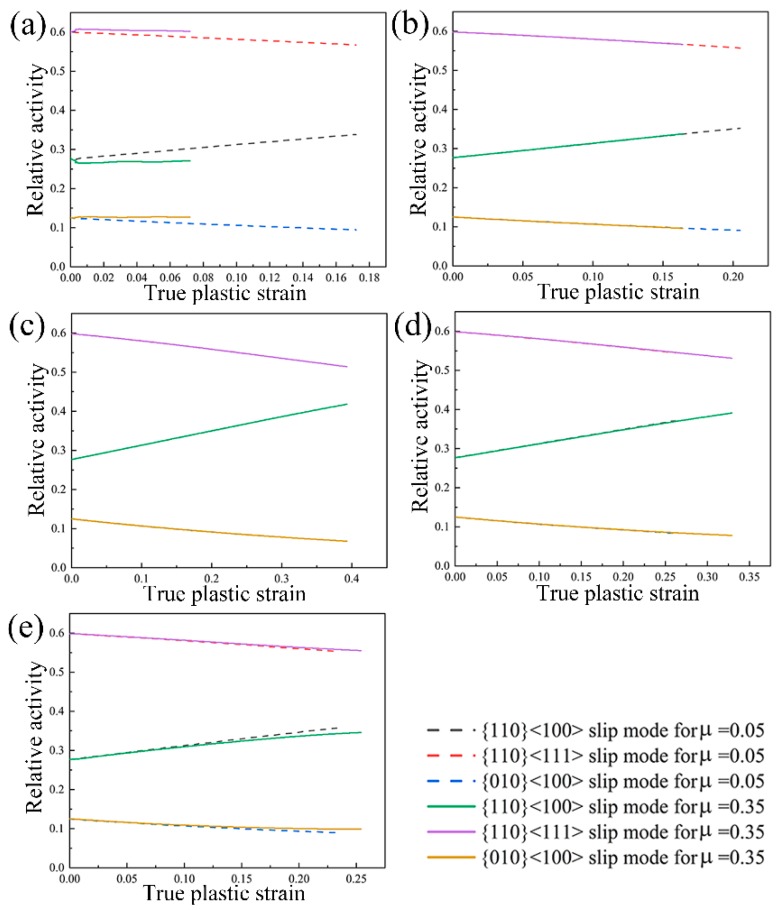
Predicted slip mode activities in the case of friction coefficient of 0.05 and 0.35 during uniaxial compression at the deformation degree of 20%: (**a**) In No. 6338 element; (**b**) In No. 5551 element; (**c**) In No. 3598 element; (**d**) In No. 3555 element; (**e**) In No. 3528 element. (The symbol μ stands for the friction coefficient.).

Due to the aforementioned investigations, it can be concluded that the friction influences the velocity gradient within the deformed sample and further contributes to the texture inhomogeneity during plastic deformation. It is well known that the deformation mechanisms have a significant influence on the texture evolution of the deformed sample. Therefore, it is of great importance to investigate the deformation mechanisms during uniaxial compression of NiTi shape memory alloy in consideration of friction effect. The slip mode activities with respect to {110} <100> slip mode, {110} <111> slip mode and {010} <100> slip mode during uniaxial compression at the deformation degree of 20% are all shown in Figure 10. It can be seen from Figure 10 that with respect to No. 6338 element, on the one hand, the slip mode activities almost keep constant during plastic deformation in the case of friction coefficient of 0.35, as compared to the ones in the case of friction coefficient of 0.05. On the other hand, by comparison with the suffered plastic strain in all three slip modes in the case of friction coefficient of 0.05, all three slip modes sustain less plastic strain in the case of friction coefficient of 0.35. This observation contributes to the difference in texture evolution with the friction coefficient of 0.05 and 0.35. Except for No. 6338 element in the minimum deformation zone, other selected elements show relatively similar evolution laws in three slip modes. The predicted activity of {110} <100> slip mode increases with the plastic strain, while the predicted activities of {110} <111> slip mode and {010} <100> slip mode decrease with the plastic strain. In addition, it is interesting to find that the evolution rates with respect to {110} <100> slip mode and {110} <111> slip mode are fastest for No. 3598 element in the principal deformation zone as compared to other selected elements. This observation is associated with the intensity of <111> fiber texture component during uniaxial compression shown in Figure 7 and Figure 9. 

## 4. Conclusions

In the present study, the coupled finite element modeling via ABAQUS code and crystal plasticity modeling via VPSC model are applied to shed a light on the friction effect on texture evolution during uniaxial compression of NiTi shape memory alloy with the assistance of dislocation slip. The following conclusions can be drawn.

1. By using the PSO algorithm in parameter calibration, the material parameters used in VPSC model are identified with efficiency and accuracy. In addition, among the accessible linearization schemes in VPSC model, the affine scheme and Neff = 10 scheme give the best predictions.
2. The friction effect has an obvious influence on the plastic deformation in the minimum deformation zone close to the dies and further affects the corresponding texture evolution during uniaxial compression. In the intermediate deformation zone and the principal deformation zone, the friction effect on the velocity gradient rapidly decrease and therefore has a minor influence on the texture evolution.
3. In the minimum deformation zone close to the dies, the friction coefficient not only affects the evolution laws with respect to the slip mode activities but also the suffered plastic strain. Whereas in the intermediate deformation zone and the principal deformation zone, there exist relatively similar evolution laws with respect to the slip mode activities, but the evolution rates with respect to {110} <100> slip mode and {110} <111> slip mode are fastest in the principal deformation zone, which contributes to the change of intensity with respect to <111> fiber texture component.

## Figures and Tables

**Table 1:** Material parameters of the adopted NiTi shape memory alloy used in VPSC model.

**Material Parameter**	$\tau_{0}^{}$	$\tau_{1}^{}$	$\theta_{0}^{}$	$\theta_{1}^{}$	${\dot{\gamma }}_{0}$	m
**Parameter Value**	174.80 MPa	465.13 MPa	5173.25 MPa	5.30 MPa	0.001 s^−1^	0.05

**Figure A1:**
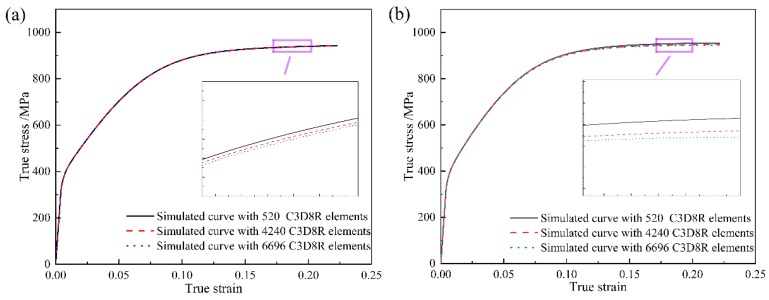
Simulated stress-strain curves in the case of 520 C3D8R elements, 4240 C3D8R elements and 6696 C3D8R elements during uniaxial compression with various coefficient of friction: (**a**) Friction coefficient of 0.05; (**b**) Friction coefficient of 0.35.

## Appendix A

The convergence study with respect to the mesh density is shown in Figure A1. The specimen of NiTi shape memory alloy has been meshed with 520 C3D8R elements, 4240 C3D8R elements and 6696 C3D8R elements, respectively. The predicted stress-strain curves during uniaxial compression are very akin to each other in the case of friction coefficient of 0.05 and 0.35. Furthermore, these predicted mechanical responses are all convergent to the ones with 6696 C3D8R elements. Therefore, the refined mesh with 6696 C3D8R elements is applicable in the present study.
